# Difference of serum tumor markers in different clinical stages of elderly patients with non-small cell lung cancer and evaluation of diagnostic value

**DOI:** 10.5937/jomb0-39798

**Published:** 2023-10-27

**Authors:** Wen Wen Qin, Ping Wang, CuiMin Ding, Fei Peng

**Affiliations:** 1 The Fourth Hospital of Hebei Medical University, Department of Respiratory Medicine, Shijiazhuang City, China

**Keywords:** elderly non-small cell lung cancer, serum tumor markers, clinical staging, diagnostic value, stariji nesitnoćelijski karcinom pluća, serumski tumor markeri, kliničko postavljanje, dijagnostička vrednost

## Abstract

**Background:**

To explore the difference and diagnostic value evaluation of serum tumor markers in different clinical stages of elderly non-small cell lung cancer (NSCLC) patients.

**Methods:**

Select 100 elderly NSCLC patients admitted to our hospital from June 2018 to June 2021, collect the general data, pathology data and imaging data of the patients, and the patients were divided into I-IV clinical stages according to the International Union Against Cancer (UICC) 8th edition lung cancer TNM staging standard. Detect the subjects' peripheral serum tumor markers, serum carcinoembryonic antigen (CEA), cytokeratin fragment 21-1 (CYFRA21-1), squamous cell carcinoma-associated antigen (SCCA), carbohydrate antigen 125 (CA125) and sugar Class antigen 199 (CA199). Compare the differences of serum CEA, CYFRA21-1, SCCA, CA125, CA199 levels in different clinical stages of elderly NSCLC patients, and the diagnostic value of the above indicators for elderly NSCLC patients was analyzed by receiver operating characteristic curve (ROC curve) and area under the curve (AUC).

## Abstract (sr)

**Uvod:**

Cilj je bio da se istraži razlika i proceni dijagnostička vrednost serumskih tumorskih markera u različitim kliničkim stadijumima starijih pacijenata sa karcinomom ne-malih ćelija pluća (NSCLC).

**Metode:**

Odabrano je 100 starijih pacijenata sa NSCLC koji su primljeni u na{u bolnicu od juna 2018. do juna 2021. godine, prikupljeni su opšti podaci, podaci o patologiji i podaci o slikama pacijenata, a pacijenti su podeljeni u I-IV kliničke stadijume prema Međunarodnoj uniji za borbu protiv raka (UICC) 8. izdanje TNM standarda za stadijum raka pluća. Otkriveni su periferni tumorski markeri u serumu ispitanika, serumski karcinoembrionalni antigen (CEA), fragment citokeratina 21-1 (CIFRA21-1), antigen povezan sa karcinomom skvamoznih ćelija (SCCA), antigen ugljenih hidrata 125 (CA125) i antigen klase šećera (CA199). Upoređene su razlike u serumskim nivoima CEA, CIFRA21-1, SCCA, CA125, CA199 u različitim kliničkim stadijumima starijih pacijenata sa NSCLC, a dijagnostička vrednost gore navedenih indikatora za starije pacijente sa NSCLC analizirana je karakterističnom krivom (ROC kriva) i površina ispod krive (AUC).

**Rezultati:**

Istorija pušenja, stepen diferencijacije, metastaze u limfnim čvorovima i prečnik tumora kod starijih pacijenata sa NSCLC sa različitim kliničkim stadijumima značajno se razlikuju; sa povećanjem progresije NSCLC bolesti, nivoi serumskih CEA, CIFRA21-1, SCCA, CA125 i CA199 nastavljaju da rastu, u serumu. Postoji značajna korelacija između markera; serumski CEA, CIFRA21-1, SCCA, CA125, CA199 imaju dijagnostičku vrednost za kliničko stadijume starijih pacijenata sa NSCLC.

**Zaključak:**

Povišenje serumskih CEA, CIFRA21-1, SCCA, CA125 i CA199 kod starijih pacijenata sa NSCLC je u pozitivnoj korelaciji sa stepenom maligniteta tumora. Otkrivanje navedenih indikatora je od velike vrednosti za ranu dijagnozu i praćenje bolesti kod starijih NSCLC.

## Introduction

Non-small cell lung cancer (NSCLC) is one of the most common types of lung cancer, accounting for about 80% of lung cancer patients, and its incidence is closely related to factors such as air pollution, smoking, and ionizing radiation [1] [2] [3]. Although advanced early screening technology and diagnosis and treatment methods have improved the efficiency of diagnosis and treatment of NSCLC, most patients have progressed to the middle and late stage when they see the doctor, miss the optimal operation time, and have poor surgical tolerance [4] [5] [6]. According to statistics, the 5-year survival rate of NSCLC is extremely low, only about 20%, and the majority of deaths are elderly people over 60 years old [3] [7] [8] [9]. At present, although it is clinically possible to treat elderly NSCLC patients by means of surgery or chemotherapy, in order to achieve the purpose of clearing the disease and delaying the life cycle of the patient [10] [11]. However, some patients may still have recurrence and metastasis after undergoing surgery or radiotherapy and chemotherapy, and the prognosis effect is not ideal [12] [10].

With the in-depth research of molecular genetics and molecular biology, domestic and foreign scholars have gradually gained a deeper understanding of the etiology and pathogenesis of NSCLC [13] [14] [15]. Many carcinogenic factors can cause cell transformation and carcinogenesis by inactivating tumor suppressor genes or activating proto-oncogenes [16] [17]. Nowadays, actively searching for effective molecular markers has very positive significance for early diagnosis, targeted therapy and prognosis judgment [18]. Serum tumor marker detection has the advantages of simple operation and economy [19] [20]. It is an important substance used in clinical to distinguish tumor cells from normal tissue cells, and become a research hotspot. Among them, serum carcinoembryonic antigen (CEA), cytokeratin fragment 21-1 (CYFRA21-1), squamous cell carcinoma associated antigen (SCCA), carbohydrate antigen 125 (CA125) and carbohydrate antigen 199 (CA199) are common tumor markers. This study analyzed the differences in serum levels of CEA, CYFRA21-1, SCCA, CA125, and CA199 in different clinical stages of elderly NSCLC patients, and evaluated their diagnostic value for elderly NSCLC patients through receiver operating characteristic curve (ROC curve) and area under the curve (AUC).

## Materials and methods

### General information

A total of 100 elderly patients with NSCLC who were admitted to our hospital from June 2018 to June 2021 were selected. Inclusion criteria: (1) confirmed by imaging and pathological examinations; (2) age greater than 60 years old; (3) no anti-tumor therapy such as radiotherapy or chemotherapy; (4) clinical data and follow-up data are complete. Exclusion criteria: (1) combined with other malignant tumors, (2) combined with severe damage to important organs such as heart, liver and kidney; (3) combined with physical diseases; (4) combined with autoimmune diseases; (5) combined with mental diseases. This study was approved by the The Fourth Hospitalof Hebei Medical University ethics committee, and all subjects gave informed consent and signed aninformed consent form(2022KS018).

### Clinical information

According to the International Union Against Cancer (UICC) 8th edition lung cancer TNM staging standard [21], all patients are divided into I-IV clinical stages; general data of patients (including age and gender) are collected and whether they have a history of smoking; the pathological type is determined to be adenocarcinoma or squamous cell carcinoma through histopathological examination; it is divided into poorly differentiated and well-differentiated according to the degree of differentiation; needle biopsy is used to assess whether lymphatic metastasis has occurred; tumor diameter is detected by imaging.

### Detection of serum tumor markers

Collect 5 mL of fasting venous blood from all subjects in the morning, and centrifuge at 3000 r/min for 10 min to obtain serum. Chemiluminescence immunoassay analyzer (Roche, Sweden) was used to detect serum levels of CEA, CYFRA21-1, SCCA, CA125, and CA199. All operations are carried out in strict accordance with the instructions.

### Statistical analysis

All data are statistically analyzed using SPSS 22.0 software (SPSS, Inc., Chicago, IL, USA). The count data is expressed as N and the chi-square test is performed, and the measurement data is expressed as mean ± standard deviation and a one-way analysis of variance is performed. GraphPad 8.0 software (GraphPad software Inc., La Jolla, CA, USA) was used for drawing. *p* <0.05 is considered statistically different.

## Results

### General clinical data

Among all 100 elderly NSCLC patients, 13 were in stage I, 25 were in stage II, 27 were in stage III, and 35 were in stage IV. Among them, stage I patients are 60–83 years old, with an average age of (71.20±5.92) years; 6 males, 7 females, 3 people have a history of smoking; pathological type: 7 cases of adenocarcinoma, 6 cases of squamous cell carcinoma; differentiation degree: 5 cases were highly differentiated, 8 cases were poorly differentiated; 4 cases of lymph node metastasis; tumor diameter: 5 cases of 5 cm, 8 cases of less than 5 cm; stage II patients are 60–82 years old, with an average (71.10±5.93) years; 12 males, 13 females, 11 people have a history of smoking; pathological types: 14 cases of adenocarcinoma, 11 cases of squamous cell carcinoma; differentiation degree: 8 cases of highly differentiated, 17 cases were poorly differentiated; 7 cases of lymph node metastasis; tumor diameter: 7 cases of 5 cm, 18 cases of less than 5 cm; stage III patients are 62-79 years old, with an average age of (71.01±5.98) years; 18 males, 9 females, 17 have a history of smoking; pathological types: 12 cases of adenocarcinoma, 15 cases of squamous cell carcinoma; differentiation degree: 16 cases were highly differentiated, 11 cases were poorly differentiated; 18 cases of lymph node metastasis; tumor diameter: 17 cases of 5 cm, 10 cases of less than 5 cm; stage IV patients are 64–80 years old, with an average (71.15±5.95) years old; 22 males, 13 females, 22 have a history of smoking; pathological types: 19 cases of adenocarcinoma, 16 cases of squamous cell carcinoma; differentiation degree: 23 cases of highly differentiated, 12 cases of poorly differentiated; 17 cases of lymph node metastasis; tumor diameter: 23 cases of 5 cm, 12 cases of less than 5 cm. There was no statistical difference in age, gender, and pathological type of patients in each stage, but there were significant differences in smoking history, degree of differentiation, lymph node metastasis, and tumor diameter (Table 1).

**Table 1:** Comparison of clinical data of elderly NSCLC patients *p < 0.05

	Stage I<br>(n=13)	Stage II<br>(n=25)	Stage III<br>(n=27)	Stage IV<br>(n=35)	*P* value
Age	71.20±5.92	71.10±5.93	71.01±5.98	71.15±5.95	0.3238
Gender					0.3999
male	6	12	18	22	
female	7	13	9	13	
Smoke					0.0475*
yes	3	11	17	22	
no	10	14	10	13	
Pathological type					0.8330
adenocarcinoma	7	14	12	19	
squamous cell carcinoma	6	11	15	16	
Differentiation degree					0.0427*
highly differentiated	5	8	16	23	
poorly differentiated	8	17	11	12	
Lymph node metastasis					0.0266*
yes	4	7	18	17	
no	9	18	9	18	
Tumor diameter					0.0137*
≥5cm	5	7	17	23	
<5cm	8	18	10	12	

### Serum tumor markers

**Figure 1:**
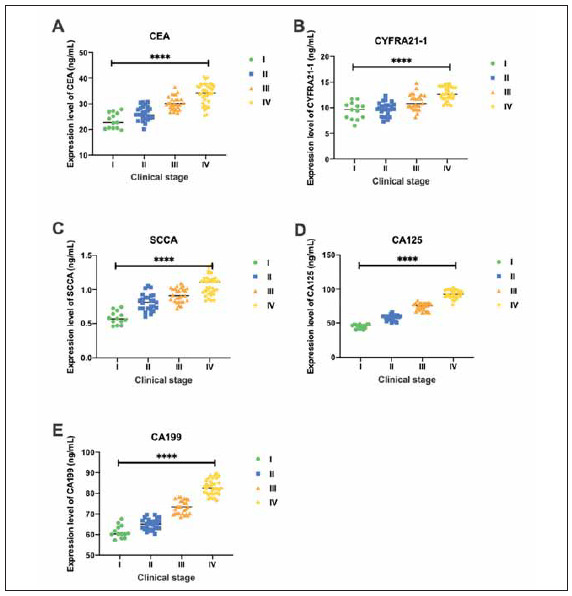
The expression levels of serum tumor markers in various clinical stages of elderly NSCLC patients. (A) The expression level of serum CEA in each clinical stage of elderly NSCLC patients; (B) The expression level of serum CYFRA21-1 in each clinical stage of elderly NSCLC patients; (C) The expression level of serum SCCA in each clinical stage of elderly NSCLC patients; (D) The expression level of serum CA125 in each clinical stage of elderly NSCLC patients; (E) The expression level of serum CA199 in each clinical stage of elderly NSCLC patients. ****p<0.0001

In order to study the expression patterns of serum tumor markers in elderly NSCLC patients at various clinical stages, we tested the serum levels of CEA, CYFRA21-1, SCCA, CA125 and CA199 in all patients. The results showed that the serum levels of CEA, CYFRA21-1, SCCA, CA125 and CA199 gradually increased with the patient's disease progression, suggesting that as the NSCLC disease progression increases, the serum tumor marker levels are also increasing (Figure 1A-E).

**Figure 2:**
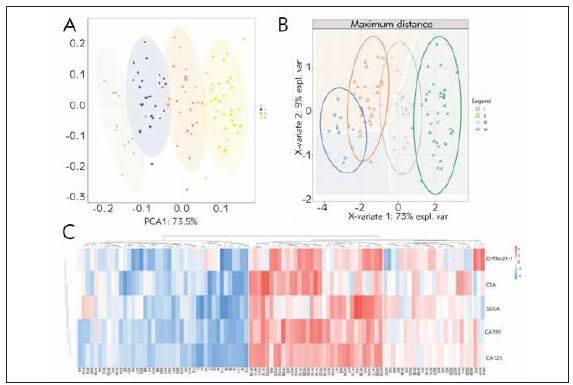
The expression pattern of serum tumor markers in various clinical stages of elderly NSCLC patients. (A) PCA analysis of serum tumor markers in elderly NSCLC patients; (B) PLS-DA analysis of serum tumor markers in elderly NSCLC patients; (C) Cluster heat map of tumor markers in various clinical stages of elderly NSCLC patients

**Figure 3:**
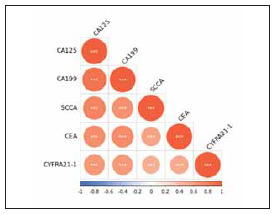
Correlation analysis of serum tumor markers in elderly NSCLC patients. ***p<0.001

Unsupervised PCA analysis was performed with serum tumor markers, and the results showed that the samples of each clinical stage were significantly separated between the PC1 dimension groups (Figure 2A); at the same time, the supervised PLS-DA discriminant analysis with serum tumor markers also showed that the clinical staging sample groups tended to cluster, and the groups tend to be dispersed (Figure 2B), suggesting that the clinical staging samples of elderly NSCLC patients are similar within the sample groups, and there are significant differences between the groups. The cluster analysis heat map showed that the phase I-II and phase III-IV of elderly NSCLC patients were clearly separated, suggesting that the serum tumor markers CEA, CYFRA21-1, SCCA, CA125 and CA199 can distinguish the clinical stages of elderly NSCLC patients (Figure 2C). Further correlation analysis of serum tumor markers in elderly NSCLC patients showed that there was a significant correlation between serum CEA, CYFRA21-1, SCCA, CA125 and CA199 (Figure 3).

### The diagnostic value of serum tumor markers in elderly patients with NSCLC

**Figure 4:**
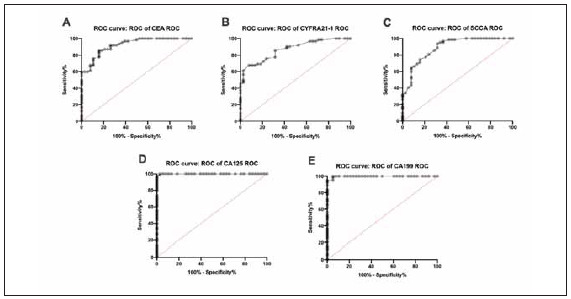
The diagnostic value of serum tumor markers in elderly patients with NSCLC. (A) The diagnostic value of serum CEA in elderly NSCLC patients; (B) The diagnostic value of serum CYFRA21-1 in elderly NSCLC patients; (C) The diagnostic value of serum SCCA in elderly NSCLC patients; (D) The diagnostic value of serum CA125 in elderly NSCLC patients (E) The diagnostic value of serum CA199 in elderly patients with NSCLC

The clinical stages of elderly NSCLC patients were divided into I-II and III-IV stages. The diagnostic value of serum CEA, CYFRA21-1, SCCA, CA125 and CA199 in elderly NSCLC patients was analyzed by ROC curve and AUC. The results showed that the AUC of serum CEA, CYFRA21-1, SCCA, CA125 and CA199 were 0.9217, 0.8680, 0.8888, 0.9994, 0.9975, respectively, and the 95% confidence intervals were 0.8716–0.9718, 0.8007–0.9353, 0.8222–0.9552, 0.9975-1, and 0.9926-1, suggestingthat serum tumor markers have diagnostic value for elderly NSCLC patients (Figure 4A-B).

## Discussion

The onset of elderly NSCLC is insidious and lacks typical clinical phenotypes in the early stage [22] [23] [24]. When patients have symptoms such as dyspnea and coughing, their condition often progresses to the middle and late stages. Imaging screening such as chest X-ray, CT, magnetic resonance and PET-CT are commonly used clinical tumor detection methods, but due to their respective limitations, they are not suitable for early screening of NSCLC [25] [26]. Furmore, In some studies, metabolomic analysis of lung SCC and AC provides a new index for the differential diagnosis of NSCLC [27]. Another interesting field of investigation concerns the development of prognostic models and immunoscoring strategies to stratify early stage patients [28].There are also studies that use the combined targeted DNA and RNA sequencing with an ion semiconductor sequencing platform to detect conventional molecules to diagnose middle and advanced NSCLC [29]. However, the above research methods adopt relatively new technical means to diagnose NSCLC, but the sample collection and operation techniques involved in these methods are relatively complex, and have no obvious advantages for early NSCLC screening. However, in our study, the use of serum tumor markers has the advantages of good repeatability, small trauma, and convenient operation. In addition, the correlation analysis of tumor staging and tumor markers has diagnostic value for the clinical stage of early NSCLC patients. One of the main reasons for the high mortality of NSCLC is the untimely diagnosis and treatment of the elderly. Therefore, the identification of new tumor markers for elderly NSCLC and the improvement of early diagnosis are essential to improve the overall survival rate.

Serum tumor marker is a peptide substance that is produced or secreted and released into the periphery during the host body's tumorigenesis and development [19] [30]. Its serum detection can reflect the characteristics and stages of the transformation process of tumor cells, and is used in the early diagnosis of tumor diseases [20]. The application value of the judgment of curative effect and prognosis evaluation is relatively high [31]. The detection of serum tumor markers has the advantages of simple operation and good repeatability. However, clinical practice shows that there may be different markers in the same tumor, the same tumor markers may appear in different pathological types of the same tumor, or multiple different tumor markers may appear. This study explores the value of serum CEA, CYFRA21-1, SCCA, CA125 and CA199 in the diagnosis of elderly NSCLC risk prediction, in order to provide reference value for clinical diagnosis of elderly NSCLC.

The degree of tumor malignancy is the proliferation and invasion activity of tumor cells, which is mainly determined by the expression of related proliferation/invasion genes [32]. TNM staging is jointly determined by the primary tumor, lymph nodes, and distant metastasis [33]. Generally speaking, patients with high TNM staging have a higher degree of tumor malignancy, but the specific proliferation and invasion gene expression is the most reliable way to prove its internal connection. Tumor recurrence and metastasis are important factors affecting the prognosis of elderly NSCLC. The killing of cancer cells by radiotherapy or chemotherapy and targeted drugs can delay the recurrence and metastasis of tumors on the basis of reducing tumor foci, thereby improving the long-term prognosis of the disease. CEA, CYFRA21-1, SCCA have been proven to be serum tumor markers for early diagnosis of lung cancer [34] [35]. CEA is an acid glycoprotein produced by colorectal cancer tissue. As a broad-spectrum tumor marker, CEA can not only reflect the tumor status in the patient, but also determine the treatment of breast cancer, colorectal cancer and lung cancer [5]. Efficacy, to determine the progress of the patient's condition, as well as to monitor the patient's condition and prognosis. According to statistics, about 65% of lung cancer patients have high expression of serum CEA, and patients with lung adenocarcinoma have higher serum CEA levels than patients with lung squamous cell carcinoma and small cell lung cancer. CYFRA21-1 has only a small amount of enlargement in normal bronchial squamous epithelium, while in malignant squamous epithelium, the activation of proteases accelerates the separation and degradation of cytokeratin, resulting in a large amount of cytokeratin fragments being released into the blood, resulting in an abnormal increase in serum CYFRA21-1 levels [36] [37] [38]. Previous reports have shown that it is mainly expressed in squamous carcinoma cells, but also in adenocarcinoma and small cell lung cancer. SCCA is a glycoprotein derived from squamous cell carcinoma. It is produced during normal and malignant transformation and is regulated by proteolysis [39]. It is one of the specific markers for the diagnosis of squamous cell carcinoma. Both CA125 and CA199 are glycoprotein antigens that are abnormally expressed in tumors [40]. Among them, CA125 is a specific tumor marker for ovarian cancer [41] [42]. In recent years, studies have found that its expression is also abnormally increased in lung cancer patients, which is related to the increase in tumor burden. In lung cancer patients with pleural fluid, the secretion of pleural mesothelial cells can be increased due to pleural fluid stimulation; CA199 is an oligomeric carbohydrate mucin. It was originally found in colon cancer tissues. It is mainly used as a tumor marker for colorectal cancer or pancreatic cancer [43]. The increase in its level also suggests diseases such as pancreatitis, liver cirrhosis, diabetes, or gastrointestinal tumors, and then there are abnormal expressions of CA199 in other tumors. In this study, serum CEA, CYFRA21-1, SCCA, CA125 and CA199 levels are different in different stages of elderly NSCLC patients, and they increase with the severity of the disease, suggesting that it may be an effective indicator for the diagnosis of elderly NSCLC.

## Conclusion

In summary, the elevation of serum CEA, CYFRA21-1, SCCA, CA125 and CA199 in elderly patients with NSCLC is positively correlated with the degree of tumor malignancy. The detection of the above indicators is of great value for the early diagnosis and condition monitoring of elderly NSCLC.

## Dodatak

### Acknowledgments

Not applicable.

### Funding

Not applicable.

### Ethical approval statement

This study was approved by the The Fourth Hospital of Hebei Medical University ethics committee, and all subjects gave informed consent and signed aninformed consent form (2022KS018).

### Conflict of interest statement

All the authors declare that they have no conflict of interest in this work.
